# Measurability of D-concurrence

**DOI:** 10.1038/s41598-019-54247-2

**Published:** 2019-12-19

**Authors:** N. Karimi, A. Heshmati, M. Yahyavi, M. A. Jafarizadeh, A. Mohammadzadeh

**Affiliations:** 1 Department of science, Farhangian University, Tehran, 19989-63341 Iran; 2 Department of Physics, Shabestar Branch, Islamic Azad University, Shabestar, Iran; 3 Department of Theoretical Physics and Astrophysics, University of Tabriz, Tabriz, 51664 Iran

**Keywords:** Mathematics and computing, Physics

## Abstract

An effective approach to quantify entanglement of any bipartite systems is D-concurrence, which is important in quantum information science. In this paper, we present a direct method for experimental determination of the D-concurrence of an arbitrary bipartite pure state. To do this, we show that measurement of the D-concurrence of bipartite pure state can be conversed into the measurement performed on some observables so called generalized Gell-Mann operators. We first introduce the concept of D-concurrence for a bipartite system. Then we explain the method of measuring this entanglement measure for the pure state. Finally, for clarify of the subject, we give an example consisting of two parties A and B with dimensions 3.

## Introduction

One of the weirdest features of the quantum world is entanglement of particles that describes the correlation of fundamental properties that cannot happen by chance. It occurs when pairs or groups of particles interact such that the quantum state of each particle cannot be described independently of the state of the others, even though the individual particles may be spatially separated. Quantum entanglement has applications in the emerging technologies of quantum computing^1^ and quantum cryptography^2^, and quantum teleportation^3^. Quantifying an amount of entanglement of a given state is defined as an entanglement measure. The first step in finding the entanglement measure for a given pure multipartite state was made by Bennett *et al*.^4^. They found that the partial entropy of a party in a bipartite quantum state can be a measure of entanglement. Next, the exact formula for the entanglement of formation for all mixed states of two qubits that have not more than two non-zero eigenvalues was obtained by Hill and Wootters^5^. Also in^6^, an explicit formula for the entanglement of formation of a pair of qubits as a function of their density matrix is obtained. They^5,6^ expressed the entanglement of formation of a two-qubit state in terms of the concurrence. The concurrence as an entanglement measures for a given state is zero iff the state is separable and it is equal to one for the maximal entangled states. It is defined for two-qubit pure and mixed states. In^7^, some direct concurrence measurement protocols are explained for the optical and atomic states such that these protocols encode the concurrence into the success probability for picking up the balanced state. For the higher dimensional bipartite states, Ma *et al*.^8^ defined a new entanglement measure that is called D-concurrence that has deep connection with the concurrence. In^9^, some of new upper and lower bounds of D-concurrence of the compound states are obtained. On the other hand, because of some unphysical quantum operation in the definition of the entanglement measures, such as complex conjugation in the concurrence^6^, in order to directly measure the entanglement of any quantum state, there isn’t measurable observable. If we can write the density matrix of a quantum state in terms of observables that are measurable, then we can experimentally determine the entanglement. This method is called tomography of the quantum state^10^. But this is not always practical, especially in the systems with the higher dimensions, since in these cases, the observables needed to measure the entanglement increase rapidly. Observables such as Pauli operators can be used for measuring the concurrence of two qubit pure states as an entanglement measure^11^. Zhou *et al*.^12^ found an efficient way for measuring the concurrence of the entanglement atoms using single photons. Sheng *et al*.^13^ showed that measuring the concurrences for polarization and momentum entanglements can be measured directly and proposed an way for measuring the concurrence for the hyperentanglement. The approaches of the concurrence measurement in both a linear and a nonlinear optical system and some ways for measuring the concurrence of the atomic entanglement system were introduced by Zhou and Sheng in^7^. The direct method for determining the negativity of an two-qubit state as an entanglement measure using relation between the purity, negativity and a universal entanglement witness was described by Bartkiewicz *et al*.^14^. In^15^, Tukiainen *et al*. proposed a protocol to quantify the concurrence of any two-qubit pure state using weak measurements and weak values. Also, a direct method for measuring quantum entanglement of arbitrary two-qubit states through Hong-Ou-Mandel interference has been described by Bartkiewicz *et al*. in^16^. In^17^, Walborn *et al*. proposed a protocol in which the concurrence of any pure quantum state can be experimentally determined by a simple projective measurement provided one has access to twofold copy of the state. Also, in^18^, Zhang *et al*. presented three schemes for directly measuring the concurrence of two-photon polarization-entangled pure and mixed states. In the case of measurability of the entanglement measures for the quantum systems with more than two-qubit, in^19^ we obtained the measurability of the polynomial invariant of degree 2 for even- N qubit pure states^20^. Given these papers and the importance of the measurability of the entanglement measures, has led us to investigate the measurability of one of the entanglement measures, so called, D-Concurrence in paper. In this paper, we give a physical interpretation of D-concurrence of an arbitrary *d* dimensional pure state and show that the measuring of the D-concurrence of any bipartite pure state can be conserved into measuring of generalized Gell-Mann operators.

The organization of this paper is as follows: In the first section of this paper, we review the concept of D-concurrence for a bipartite system. In section 3, we explain a direct method of measuring this en- tanglement measure for bipartite pure state. In section 4, for clarify of the subject, we give an example consisting of two parties A and B with dimension 3. Finally, we conclude the paper in the last section.

## D-concurrence

Consider the system consists of two parties A and B with corresponding state spaces of *H*_*A*_ and *H*_*B*_. The state of the system is $|\psi \rangle \in {H}_{A}\otimes {H}_{B}$. If the dimension of ${H}_{A}\otimes {H}_{B}$ is finite, then the D-concurrence of the system is given by:

2.1 $$C(\psi )=\sqrt{2[1-Tr({\rho }_{A}^{2})]}$$

which *ρ*_*A*_ represents the reduce density matrix, i.e., ${\rho }_{A}=T{r}_{B}(|\psi \rangle \langle \psi |)$. This definition is extended to the mixed states by means of the convex roof extension. Mathematically, for the mixed state $\rho =\sum _{i}{p}_{i}|\psi \rangle \langle \psi |$ with $\sum _{i}{p}_{i}=1$, the D-concurrence is defined as

2.2 $$C(\rho )={\min }_{\{{p}_{i},|{\psi }_{i}\rangle \}}\sum _{i}{p}_{i}C({\psi }_{i})$$

which $\{{p}_{i},|{\psi }_{i}\rangle \}$ is the ensemble of a pure state for the given density matrix. The ensemble that minimizes *C*(*ρ*) is called optimal^21^.

On the other hand for the infinite dimensional of the Hilbert space ${H}_{A}\otimes {H}_{B}$, it can be shown that if the Schmidt decomposition of $|\psi \rangle$ is as $|\psi \rangle =\sum _{k}{\lambda }_{k}|k\rangle |k^{\prime} \rangle$, then^22^

2.3 $$C(\psi )=2\sqrt{\sum _{k\ne l}{\lambda }_{k}^{2}{\lambda }_{l}^{2}}.$$

The proof of the above equation is as follows: For this state $|\psi \rangle$, the reduce density matrix *ρ*_*A*_ is the diagonal matrix with the elements as ${\rho }_{A}=Diag\{{\lambda }_{1}^{2},\,{\lambda }_{2}^{2},\mathrm{..}.,{\lambda }_{k}^{2}\}$. Given that ${\lambda }_{1}^{2}+{\lambda }_{2}^{2}+\mathrm{..}.+{\lambda }_{k}^{2}=1$ and using Eq. (2.1), we have:

2.4 $$\begin{array}{ccc}C(\psi ) & = & \sqrt{2[{({\lambda }_{1}^{2}+{\lambda }_{2}^{2}+\mathrm{..}.+{\lambda }_{k}^{2})}^{2}-({\lambda }_{1}^{4}+{\lambda }_{2}^{4}+\mathrm{..}.+{\lambda }_{k}^{4})]}\\  & = & 2\sqrt{\sum _{k\ne l}{\lambda }_{k}^{2}{\lambda }_{l}^{2}}\end{array}$$

In the following section, we describe a direct method to determine the D-concurrence of an arbitrary bipartite pure state in experiments.

## Measurability of D-concurrence

In this paper, we want to investigate the physical interpretation of the D-concurrence defined by Eq. (2.4) that makes to measure of it directly. To do this, we use the definition of the total variance given in^10^

3.5 $$V(\psi )=\sum _{\alpha }(\langle \psi |{\Lambda }_{\alpha }^{2}|\psi \rangle -{\langle \psi |{\Lambda }_{\alpha }|\psi \rangle }^{2})$$

which ${\Lambda }_{\alpha }$ are the local observables so that

3.6 $${\Lambda }_{\alpha }={\Lambda }_{i}^{A}\otimes I\,or\,{\Lambda }_{\alpha }=I\otimes {\Lambda }_{j}^{B}$$

that ${\Lambda }_{i}^{A}$ and ${\Lambda }_{j}^{B}$ are orthonormal bases in the space of Hermitian operators *H*_*A*_ and *H*_*B*_ respectively. They are the standard SU(N) generators (in our study N = d) that are the generalized Gell-Mann matrices (GGM). In fact they are the extensions of Pauli matrices in higher dimensions for qubits and the Gell-Mann matrices for qutrits and defined as three different kinds of matrices as follows^23^:

(i) $$\frac{d(d-1)}{2}$$ symmetric GGM
  3.7 $${\Lambda }_{s}^{jk}=|j\rangle \langle k|+|k\rangle \langle j|,\,1\le j < k\le d,$$
(ii) $\frac{d(d-1)}{2}$ antisymmetric GGM
  38 $${\Lambda }_{a}^{jk}=-\,i|j\rangle \langle k|+i|k\rangle \langle j|,\,1\le j < k\le d,$$
(iii) (d−1) diagonal GGM

3.9 $$\begin{array}{rcl}{\Lambda }^{l} & = & \sqrt{\frac{2}{l(l+1)}}(\mathop{\sum }\limits_{j=1}^{l}|j\rangle \langle j|-l|l+1\rangle \langle l+1|),\\  &  & \,1\le l\le d-1,\end{array}$$

which all GGM are Hermitian and orthogonal and form a basis. Also in Eq. (3.6), *I* is the identity operator.

Since $[{\sum }_{i=1}^{{d}^{2}-1}{\Lambda }_{i}^{2},{\Lambda }_{i}]=0$, so ${\sum }_{i=1}^{{d}^{2}-1}{\Lambda }_{i}^{2}$ forms a Casimir operator, i.e. ${\sum }_{i=1}^{{d}^{2}-1}{\Lambda }_{i}^{2}$ is proportional to identity. Therefor:

$$\sum _{\alpha }{\Lambda }_{\alpha }^{2}=\eta I\otimes I$$

which it may be rewritten as

$$\mathop{\sum }\limits_{i=1}^{{d}^{2}-1}{\Lambda }_{i}^{2}\otimes I+\mathop{\sum }\limits_{j=1}^{{d}^{2}-1}I\otimes {\Lambda }_{j}^{2}=\eta I\otimes I.$$

By tracing of the both sides of above relation, we get:

$$tr(\sum _{\alpha }{\Lambda }_{\alpha }^{2})=\eta {d}^{2}\Rightarrow 2({d}^{2}-1)d+2d({d}^{2}-1)=\eta {d}^{2}$$

and then

$$\eta =\frac{4({d}^{2}-1)}{d}$$

so:

3.10 $$\sum _{\alpha }\langle \psi |{\Lambda }_{\alpha }^{2}|\psi \rangle =\frac{4({d}^{2}-1)}{d}$$

On the other hand:

3.11 $$\sum _{\alpha }|\langle \psi |{\Lambda }_{\alpha }|\psi \rangle {|}^{2}=\sum _{i}|\langle \psi |{\Lambda }_{i}\otimes I|\psi \rangle {|}^{2}+\sum _{j}|\langle \psi |I\otimes {\Lambda }_{j}|\psi \rangle {|}^{2}$$

The first term on the right side of the above equation can be written as:

$$\mathop{\sum }\limits_{i}^{{d}^{2}-1}|\langle \psi |{\Lambda }_{i}\otimes I|\psi \rangle {|}^{2}=\sum _{i}tr{({\rho }^{A}{\Lambda }_{i})}^{2}$$

which *ρ*^*A*^ is the reduced density matrix of the subsystem *A*. It can be rewritten in terms of the diagonal reduced density matrix of ${\rho }_{D}^{A}$ by using the local unitary transformations U. That is:

$$tr({\rho }^{A}{\Lambda }_{i})=tr({U}^{\dagger }{\rho }_{D}^{A}U{\Lambda }_{i})=tr({\rho }_{D}^{A}\mathop{\underbrace{U{\Lambda }_{i}{U}^{\dagger }}}\limits_{{R}_{ij}{\Lambda }_{j}})$$

so

$${[tr({\rho }^{A}{\Lambda }_{i})]}^{2}=\sum _{jk}{R}_{ij}{R}_{ik}tr({\rho }_{D}^{A}{\Lambda }_{i})tr({\rho }_{D}^{A}{\Lambda }_{k})=\sum _{jk}{\delta }_{jk}tr({\rho }_{D}^{A}{\Lambda }_{j})tr({\rho }_{D}^{A}{\Lambda }_{k})=\sum _{j}tr{({\rho }_{D}^{A}{\Lambda }_{j})}^{2}$$

and as a result, we have:

3.12 $$\sum _{i}\,|\langle \psi |{\Lambda }_{i}\otimes I|\psi \rangle {|}^{2}=\sum _{i}\,tr{({\rho }_{D}^{A}{\Lambda }_{i})}^{2}=\sum _{i}\,{({\lambda }_{i}^{2}{({\Lambda }_{i})}_{ii})}^{2}$$

which

$${\rho }_{D}^{A}=diag({\lambda }_{1}^{2},{\lambda }_{2}^{2},\mathrm{..}.,{\lambda }_{d}^{2})$$

Similarly, one can obtain the second term on the right side of Eq. (3.11) as follows

3.13 $$\sum _{j}|\langle \psi |I\otimes {\Lambda }_{j}|\psi \rangle {|}^{2}=\sum _{j}tr{({\Lambda }_{j}{\rho }_{D}^{B})}^{2}=\sum _{j}{({\lambda }_{j}^{2}{({\Lambda }_{j})}_{ii})}^{2}$$

which ${\rho }_{D}^{B}$ is the diagonal reduced matrix of subsystem $B$ and

$${\rho }_{D}^{B}=diag({\lambda }_{1}^{2},{\lambda }_{2}^{2},\mathrm{..}.,{\lambda }_{d}^{2})$$

In summary, using the Eqs. (3.5, 3.10, 3.12, 3.13) we obtain:

3.14 $$V(\psi )=\frac{4({d}^{2}-1)}{d}-{(\sum _{i}({\lambda }_{i}^{2}{({\Lambda }_{i})}_{ii})}^{2}+\sum _{j}({\lambda }_{j}^{2}{({\Lambda }_{j})}_{ii}{)}^{2})$$

On the other hand, it can easily be shown that:

315 $$\sum _{i}{({\lambda }_{i}^{2}{({\Lambda }_{i})}_{ii})}^{2}=\sum _{j}{({\lambda }_{j}^{2}{({\Lambda }_{j})}_{ii})}^{2}=2(1-\frac{1}{d})-{C}^{2}(\psi )$$

So, finally we obtain:

3.16 $$V(\psi )=\frac{4({d}^{2}-1)}{d}-4(1-\frac{1}{d})+2{C}^{2}(\psi )=4(d-1)+2{C}^{2}(\psi )$$

Then

3.17 $$C(\psi )=\sqrt{\frac{V(\psi )-4(d-1)}{2}}$$

Thus, the amount of entanglement carried by a pure two-partite state can be determined by measurement of mean values of the basic observables.

## Example

The physically implement of the qubits, qudits and the projective measurements are the cornerstones of the quantum states measurements. To clarify the qualitative analysis of the resource requirement for the procedure and the concepts of the pervious section, in this section we investigate Eq. (3.17) for the system consisting two parties A and B whose dimensions are 3, namely, *d* = 3. For *d* = 3, eight Gell-Mann matrices are as the following form^23^:

$${\lambda }_{s}^{12}=(\begin{array}{ccc}0 & 1 & 0\\ 1 & 0 & 0\\ 0 & 0 & 0\end{array})\,{\lambda }_{s}^{13}=(\begin{array}{ccc}0 & 0 & 1\\ 0 & 0 & 0\\ 1 & 0 & 0\end{array})\,{\lambda }_{s}^{23}=(\begin{array}{ccc}0 & 0 & 0\\ 0 & 0 & 1\\ 0 & 1 & 0\end{array})$$

$${\lambda }_{a}^{12}=(\begin{array}{ccc}0 & -i & 0\\ i & 0 & 0\\ 0 & 0 & 0\end{array})\,{\lambda }_{a}^{13}=(\begin{array}{ccc}0 & 0 & -i\\ 0 & 0 & 0\\ i & 0 & 0\end{array})\,{\lambda }_{a}^{23}=(\begin{array}{ccc}0 & 0 & 0\\ 0 & 0 & -i\\ 0 & i & 0\end{array})$$

$${\lambda }^{1}=(\begin{array}{ccc}1 & 0 & 0\\ 0 & -1 & 0\\ 0 & 0 & 0\end{array})\,{\lambda }^{2}=\frac{1}{\sqrt{3}}(\begin{array}{ccc}1 & 0 & 0\\ 0 & 1 & 0\\ 0 & 0 & -2\end{array})$$

so

$$\begin{array}{rcl}\sum _{i}tr{({\rho }_{D}^{A}{\Lambda }_{i})}^{2} & = & {({\lambda }_{1}^{2}-{\lambda }_{2}^{2})}^{2}+\frac{1}{3}{({\lambda }_{1}^{2}+{\lambda }_{2}^{2}-2{\lambda }_{3}^{2})}^{2}\\  & = & {\lambda }_{1}^{4}+{\lambda }_{2}^{4}-2{\lambda }_{1}^{2}{\lambda }_{2}^{2}+\frac{1}{3}({\lambda }_{1}^{4}+{\lambda }_{2}^{4}+4{\lambda }_{3}^{2}+2{\lambda }_{1}^{2}{\lambda }_{2}^{2}-4{\lambda }_{1}^{2}{\lambda }_{3}^{2}-4{\lambda }_{2}^{2}{\lambda }_{3}^{2})\\  & = & \frac{4}{3}({\lambda }_{1}^{4}+{\lambda }_{2}^{4}+{\lambda }_{3}^{4})-\frac{4}{3}({\lambda }_{1}^{2}{\lambda }_{2}^{2}+{\lambda }_{1}^{2}{\lambda }_{3}^{2}+{\lambda }_{2}^{2}{\lambda }_{3}^{2})\\  & = & \frac{4}{3}({\lambda }_{1}^{4}+{\lambda }_{2}^{4}+{\lambda }_{3}^{4})-\frac{4}{3}({\lambda }_{1}^{2}{\lambda }_{2}^{2}+{\lambda }_{1}^{2}{\lambda }_{3}^{2}+{\lambda }_{2}^{2}{\lambda }_{3}^{2})\\  &  & +\frac{8}{3}({\lambda }_{1}^{2}{\lambda }_{2}^{2}+{\lambda }_{1}^{2}{\lambda }_{3}^{2}+{\lambda }_{2}^{2}{\lambda }_{3}^{2})-\frac{8}{3}({\lambda }_{1}^{2}{\lambda }_{2}^{2}+{\lambda }_{1}^{2}{\lambda }_{3}^{2}+{\lambda }_{2}^{2}{\lambda }_{3}^{2})\\  &  & \times \frac{4}{3}({\lambda }_{1}^{4}+{\lambda }_{2}^{4}+{\lambda }_{3}^{4}+2{\lambda }_{1}^{2}{\lambda }_{2}^{2}+2{\lambda }_{1}^{2}{\lambda }_{3}^{2}+2{\lambda }_{2}^{2}{\lambda }_{3}^{2})-4({\lambda }_{1}^{2}{\lambda }_{2}^{2}+{\lambda }_{1}^{2}{\lambda }_{3}^{2}+{\lambda }_{2}^{2}{\lambda }_{3}^{2})\\  & = & \frac{4}{3}{({\lambda }_{1}^{2}+{\lambda }_{2}^{2}+{\lambda }_{3}^{2})}^{2}-4({\lambda }_{1}^{2}{\lambda }_{2}^{2}+{\lambda }_{1}^{2}{\lambda }_{3}^{2}+{\lambda }_{2}^{2}{\lambda }_{3}^{2})\end{array}$$

since ${\sum }_{i}{\lambda }_{i}^{2}=1$, and by using Eq. (2.3), we have

$$\sum _{i}tr{({\rho }_{D}^{A}{\Lambda }_{i})}^{2}=\frac{4}{3}-2{C}^{2}(\rho )$$

similarly:

$$\sum _{j}tr{({\rho }_{D}^{B}{\Lambda }_{j})}^{2}=\frac{4}{3}-2{C}^{2}(\rho )$$

then, using Eqs. (3.10) and (3.5) we obtain:

$$V(\psi )=8+2{C}^{2}(\psi )$$

and therefore

$$C(\psi )=\sqrt{\frac{V(\psi )-8}{2}}$$

which is in fully agreement with Eq. (3.17) by putting *d* = 3.

The general qudit density matrix can be written as the following form:

$$\begin{array}{rcl}\rho  & = & \frac{1}{d}({S}_{0}I+\sum _{j,k\in \{0,1,\mathrm{..}.,d-1|j\ne k\}}(Tr[{\Lambda }_{s}^{jk}\rho ]{\Lambda }_{s}^{jk}+Tr[{\Lambda }_{a}^{jk}\rho ]{\Lambda }_{a}^{jk})+\mathop{\sum }\limits_{l=1}^{d-1}Tr[{\Lambda }^{l}\rho ]{\Lambda }^{l})\\  & \equiv  & \frac{1}{d}({S}_{0}I+\sum _{j,k\in \{0,1,\mathrm{..}.,d-1|j\ne k\}}({S}_{jk}^{{\Lambda }_{s}}{\Lambda }_{s}^{jk}+{S}_{jk}^{{\Lambda }_{a}}{\Lambda }_{a}^{jk})+\mathop{\sum }\limits_{l=1}^{d-1}{S}_{l}^{\Lambda }{\Lambda }^{l})\end{array}$$

which symbol *I* represents the unit matrix in d dimensions and *S*_0_ = 1. This density matrix is parametrized by *d*^2^ − 1 real parameters, called generalized Stokes parameters, (${S}_{jk}^{{\Lambda }_{s}},{S}_{jk}^{{\Lambda }_{a}}$ and ${S}_{l}^{\Lambda }$) that are measurable. In fact, by introducing optical qudits and describing optical components which are required for the realization of projective measurements, one can have a transition to quantum optics field^24^. This work is done in^25^ and a qualitative analysis of Gell-Mann parameters is given for a quantum three-mode Bose system. They obtained the variances of the Gell-Mann parameters for the optical field in the coherent state that determine fluctuations in the system. These variances are measurable. Similarly, we can measure the Gell-Mann operators. So by means of SU(3) symmetry, the schematic diagram of the SU(3) interferometer for measuring the Gell-Mann parameters, will be similar to the Fig. 1 in^26^.

## Conclusion

In summary, we have described the direct measurement of D-concurrence as an entanglement measure of any bipartite system. It is shown that the direct measurement of D-concurrence of any bipartite pure state can be encoded into the measuring of the some obsevables so-called generalized Gell-Mann operators. Finally, we have obtained the measurability of concurrence of bipartite pure state with dimension 3 by means of Gell-Mann matrices as an observables.
